# Septic arthritis: immunopathogenesis, experimental models and therapy

**DOI:** 10.1186/1678-9199-20-19

**Published:** 2014-05-06

**Authors:** Priscila Maria Colavite, Alexandrina Sartori

**Affiliations:** 1 Departamento de Microbiologia e Imunologia, Instituto de Biociências, Universidade Estadual Paulista (UNESP), Distrito de Rubião Júnior, s/n, Botucatu, São Paulo State, Brazil

**Keywords:** Septic arthritis, *Staphylococcus aureus*, Mice

## Abstract

Septic arthritis is an inflammatory disease of the joints that is started by an infection whose most common agent is *Staphylococcus aureus.* In this review we discuss some of the most arthritogenic bacterial factors and the contribution of innate and specific immune mechanisms to joint destruction. Special emphasis is given to the induction of experimental arthritis by *S. aureus* in mice. The improvement of therapy by association of antibiotics with down-modulation of immunity is also included.

## Introduction

Septic arthritis (SA), also called infectious arthritis, is an inflammatory disease of the joints that is started by an infectious agent. Typically, SA involves one large joint such as the knee or hip but can also affect any other joint. The general estimated incidence of this pathology in industrialized countries is about 6 cases per 100,000 persons per year, with the highest rates being found in those under 15 and over 55 years old [1]. The most important risk factor for SA is preexisting joint pathologies, especially rheumatoid arthritis or prosthetic joint surgery. In these patients, SA incidence increases to 70 per 100,000 persons [2]. SA is generally considered a secondary infection, that is, the bacterium escapes from the bloodstream and enters the surrounding tissues. A number of strategies such as endothelial attachment, transcytosis, paracytosis and bacterial transportation by professional phagocytes have been described as putative mechanisms that allow the infectious agent to disseminate from the blood to the joint or other tissues [3].

Host and bacterial factors are considered to be of pathogenic importance during SA. The initial focus of joint destruction is usually the cartilage-synovium junction, with pannus formation and subsequent cartilage and bone destruction. There is an inflammatory process characterized by a rapid recruitment of polymorphonuclear granulocytes and activated macrophages soon followed by T cells [4,5]. This process leads to irreversible loss of joint function and is associated with the production of a variety of cytokines [6,7]. The speed and accuracy of treatment are decisive for the outcome of SA. Even the delay of a few days in treatment may lead to permanent joint destruction and an increased mortality rate. The immunopathogenic process and the treatment will be explained below.

## Review

### Causative agents

The most common causative organism in both children and adult SA is *Staphylococcus aureus*[8,9]. *S. aureus* is the primary cause of bacterial arthritis in 40% of cases from England and Wales, 56% of cases from France and 37% of cases from tropical Australia [10-12].

Interestingly, the isolation of *S. aureus* from arthritis lesions increases to 80% in joint infections in patients with concurrent rheumatoid arthritis (RA) and in those with diabetes. This predominance of *S. aureus* has been mainly attributed to its arthritogenic virulence factors that will be described below. Streptococci from groups A, B, C and G are also commonly isolated from SA in immunocompromised hosts or in patients with severe gastrointestinal or genitourinary infections [13]. *Streptococcus pneumoniae*, *Escherichia coli, Proteus* sp.*, Salmonella* sp*., Serratia marcescens,* and *Neisseria* sp. have also been reported as causal agents of SA [1]. It has been estimated that no causative agent is identified in around half of the patients because the severity of this pathology requires a prompt therapy, even before the isolation of the infectious agent.

### Virulence factors related to arthritogenicity

A variety of virulence factors are associated with the ability of a pathogen to trigger SA. However, in this review, we will consider some of the ones related to *S. aureus* due to its status as the most prevalent microorganism in human SA and also the one that causes the most severe joint disease [3]. Some virulence factors are directly related to the ability of *S. aureus* to colonize the joint whereas others are related to the effect of the bacterium on host immunity. Some of these virulence elements are classified as adhesins because they allow the bacteria to adhere to certain tissues initiating the infection. Two main adhesin types have been described as responsible for the initial anchoring of *S. aureus* in the joints: the clumping factors (ClfA and B) and fibronectin-binding proteins (FnBPA and B).

ClfA is a surface protein that binds to fibrinogen and fibrin [14]. The main properties of ClfA, associated with the ability of *S. aureus* to cause disease, were established in a rat model of endocarditis. This adhesin is able to clump bacterial cells and to promote their adherence to blood clots, to plasma-conditioned biomaterials and to catheter-damaged heart valves [14-16]. The contribution of ClfA to the pathogenesis of *S. aureus* SA was evaluated in a murine model. Arthritis severity was strikingly reduced in mice intravenously infected with a ClfA mutant devoid of this molecule in comparison to mice infected with the wild-type bacteria that expressed ClfA. Additionally, previous active immunization with ClfA or passive immunization with anti-ClfA antibodies determined a less severe arthritis [17].

Collagen-binding protein is another adhesin that was originally isolated from the cell surface of *S. aureus*. This protein was able to mediate the attachment of *S. aureus* cells to cartilage [18]. The arthritogenic properties of this molecule were studied with two classes of *S. aureus* mutants. In the first class of mutants, the isolated collagen adhesin gene was inactivated while in the second mutant type the intact gene was introduced into an *S. aureus* strain that lacked the gene. The majority of the animals injected with the strain containing the gene developed arthritis whereas only a small proportion of the ones injected with the strain lacking this gene developed symptoms of the disease [19].

The fibronectin-binding proteins (FnBPs) A and B expressed by *S. aureus* recognize fibronectin, fibrinogen and elastin [20-22]. These proteins enable staphylococcal adherence and further invasion of different cell types, such as epithelial and endothelial cells, fibroblasts, and osteoblasts [23,24]. Through the formation of a fibronectin bridge to the fibronectin-binding integrin α5β1 expressed on the host cell surface, FnBPs trigger bacterial invasion [23-25]. It has been suggested that this invasion might provide a mechanism by which the staphylococci evade host defenses and avoid being killed by antibiotics.

More recently, the biofilm-forming capacity has been considered a major virulence determinant in *S. aureus* infection [26]. Biofilms are communities of bacterial cells, present on a surface, that are held together by a matrix of extracellular substances from both the bacteria and the host. Implanted medical devices have been described as the most characteristic support for these bacterial colonies [27]. It has been suggested that articular structures could also serve as a support for the growth of these bacterial communities [28]. The possible correlation between arthritogenicity of *S. aureus* strains and their ability to form biofilms is not well established and deserves a thorough investigation.

The elevated virulence of *S. aureus* compared to other infectious agents has been, at least partially, attributed to the many immune evasion strategies presented by this pathogen [29]. Some of these evasion mechanisms such as the expression of an extracellular capsule, the release of formylated peptides and the production of molecules endowed with superantigenic properties have been correlated with higher arthritogenicity. The extracellular capsule contains polysaccharides; among 11 reported capsular serotypes, types 5 (CP5) and 8 (CP8) comprise 80-85% of all clinical isolates from blood. By using CP5 mutants that did not express the capsule, Nilsson *et al*. [30], demonstrated that the presence of CP5, in comparison to its absence, leads to a higher frequency of arthritis and also to a more severe form of the disease. This higher arthritogenicity of the CP5 strain was attributed to the down-regulatory property of this structure on the ingestion and intracellular killing capacity of phagocytes. Formylated peptides are released by the bacterium allowing the recruitment of neutrophils into synovial tissue, thus contributing to joint destruction [31-34].

In addition, *S. aureus* produces and secretes a large number of enzymes and toxins that have been implicated in infectious arthritis [35]. A subset of these molecules displays superantigenic properties; that is, they possess the unique ability to activate a large number of T lymphocytes expressing certain Vβ sequences. As the human genome encodes approximately 50 TCR Vβ elements, it has been estimated that these superantigens (SAg) can activate up to 20% of the T cell pool [36]. This Vβ recognition is simultaneously associated with binding to antigen-presenting cells via MHC class II molecules. These interactions result in T cell proliferation and a substantial cytokine release by both cell types [37,38]. The contribution of SAg to SA has been clearly observed in experimental arthritis [39-41]. Even though the toxic shock syndrome toxin-1 (TSST-1) SAg has been more frequently (47%) found in the synovial fluid of patients with SA, enterotoxin C was also found in 39% of the cases [42].

### Immunopathogenesis

As stated previously, *S. aureus* is the leading cause of all cases of infectious arthritis. Therefore, the immunopathogenetic characteristics described below are all related to this etiological agent. The investigation of SA in humans is hampered by the difficulty not only of establishing the infection onset time but also of obtaining tissue samples from the different components of the joint such as the synovial membrane, the cartilage and the subchondral bone. Therefore, most of the information detailed below originated from experimental arthritis in mice infected with *S. aureus.*

It is well established that, in addition to bacterial virulence factors, the host immune response plays an important role in the joint-damaging process. Clinically, experimental arthritis occurs in both fore and hind paws and is characterized by visible erythema and edema. Histopathological analysis of swollen joints shows hypertrophy and proliferation of the synovial tissue, inflammation, pannus formation as well as cartilage and bone destruction [35].

One of the hallmarks of septic arthritis is the massive inflammation that precedes cartilage and bone erosion. The local bacterial proliferation is accompanied by a rapid recruitment of polymorphonuclear granulocytes (PMNs) and activated macrophages quickly followed by T cells [4]. The direct contribution of bacterial products to the recruitment of PMNs was demonstrated by Gjertsson *et al.*[34]. Differently from eukaryotic cells, bacteria start protein synthesis with a formyl methionine residue, thus originating formylated peptides which are potent chemoattractants for PMNs [43]. These authors demonstrated that recruitment of PMNs in the synovial tissue was much more discrete in mice infected with a mutant strain lacking the ability to produce formylated peptides.

Cytokines released from macrophages such as TNF-α, IL-1β and IL-6 have been classically indicated as the major players of the severe inflammation that precedes cartilage and bone destruction during SA. These molecules stimulate osteoclast differentiation and bone resorption in a synergistic fashion [44]. TNF-α, considered the most osteoclastogenic cytokine, activates NF-kB which in turn is associated with the survival of osteoclasts [5]. It is important to highlight, however, that these cytokines are also relevant to protect the host against the infectious agent [45]. The high significance of this protective role is illustrated by the demonstration that mice lacking the IL-1R type 1 developed a much more severe SA compared with intact controls, in response to infection with *S. aureus*[46].

In contrast with B cells that do not seem to contribute to the course of *S. aureus-*induced SA, T cells and their cytokines are clearly involved in this disease [47]. This direct contribution was initially suggested by the presence of T cells, predominantly of CD4^+^ phenotype, in the affected joints from experimentally infected mice [40]. Assays targeting T cell cytokines confirmed participation of this T cell subset, and also indicated its dualistic role. Administration of IFN-γ before or after *S. aureus* inoculation decreased mortality but also increased arthritis development [48]. IL-17 is a more recently described cytokine that is produced by T cell subsets and many innate-like T cells [49]. It is well established that this cytokine is an important mediator of rheumatoid arthritis in both, humans and mice [50]. Its role in *S. aureus-*triggered SA is, however, largely unknown. Data published by Henningsson *et al.*[51], suggest that IL-17A is more relevant in local rather than systemic host defense against *S. aureus*-induced arthritis. Our group recently reported that the variable arthritogenicity of *S.aureus* strains, isolated from biological samples, is probably related to their differential ability to induce IL-17 production [52].

As we described above, SAg can stimulate a great fraction of T cells, which is followed by their proliferation and subsequent secretion of cytokines and chemokines. The possible contribution of SAg such as TSST-1 to arthritis development was demonstrated by Abdelnour *et al*. [40,41]. Also by using a rat model, Bremell and Tarkowski [39], observed that almost all rats injected with SAg-producing *S. aureus* strains developed arthritis. On the other hand, only 20% of the rats injected with a strain lacking SAg developed the disease. One of the reasons why *S.aureus*-induced SA is considered a medical emergency is because this disease rapidly progresses to joint destruction. Contribution of metalloproteinases [53] and a rapid systemic bone resorption have been well characterized in experimental arthritis [5].

### Experimental models

Animal models are considered invaluable for studying the pathogenesis of many human diseases. Rabbits intra-articularly injected with bacteria have been, for a long time, used to study SA [54]. However, as in many other diseases where immunity plays a highly relevant role, mice were lately adopted for experimental studies. The characteristics of the murine model closely mirror changes seen in human SA, mainly with regard to the elevated frequency and severity of periarticular bone erosivity [4]. Additionally, mice are extremely versatile models. First of all, their immune system, which is similar to the human counterpart in many aspects, is particularly well characterized. There is also a plethora of mice strains lacking (knockout mice) or overexpressing (transgenic) certain genes, which enables a deeper investigation of their contribution to the pathology being studied. The general application of this model and our own experience with it will be discussed below.

Induction of experimental arthritis by *S. aureus* infections has been successful with certain mice strains as NRMI, C57BL/6, 1295 V and BALB/c [55-57]. A very important aspect is the choice of the *S. aureus* strain to be used. Even though *S. aureus* is the leading cause of infectious arthritis, not all the strains are arthritogenic. Tarkowski *et al*. [58] greatly contributed to establishing the basis of SA models. This group usually employs a bacterial strain denominated LS-1, originally isolated from a spontaneous outbreak of *S. aureus* arthritis in a mouse colony [59]. The production of the exotoxin TSST-1 contributes to arthritogenicity since mice injected with TSST-1-producing *S. aureus* strain developed more frequently and also a more severe disease than strains that do not produce this SAg [40,41]. However, we recently observed that *S. aureus* strains able to produce other SAg such as enterotoxin C and enterotoxin A were also able to trigger SA [52]. Another relevant detail is the bacterial dose, which typically ranges from 7.10^6^ to 10^7^ *S. aureus* colony-forming units per mouse [58].

**Figure 1:**
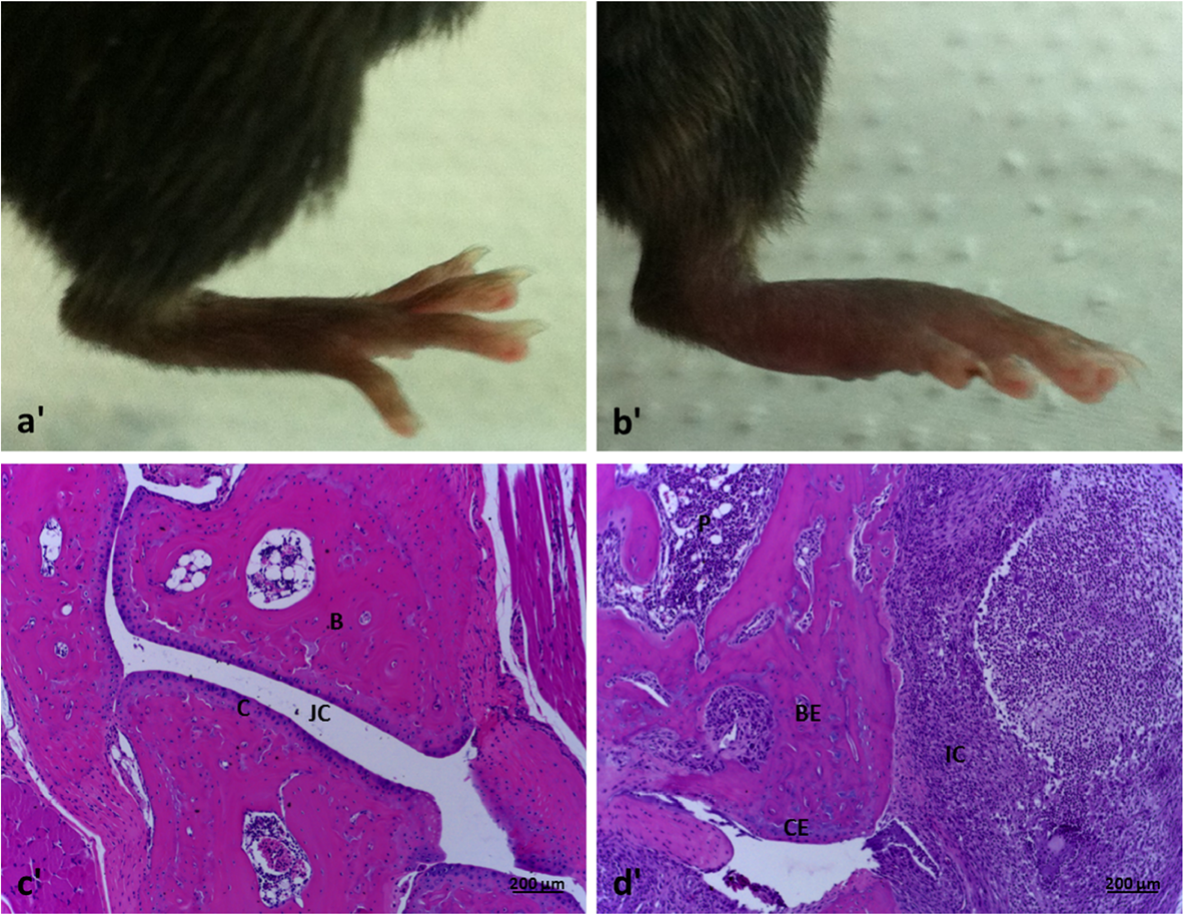
**Clinical score 2 and corresponding histopathological analyses in a hind paw from a C57BL/6 mouse infected with enterotoxin C producer *S. aureus *strain. (a’)** normal mouse; **(b’)** mouse with arthritis. **(c’, d’)** Histopathological micrographics are shown with 10x magnification. JC: joint cavity; C: cartilage; B: bone; BE: bone erosion; CE: cartilage erosion; P: pannus formation and IC: inflammatory cells.

**Figure 2:**
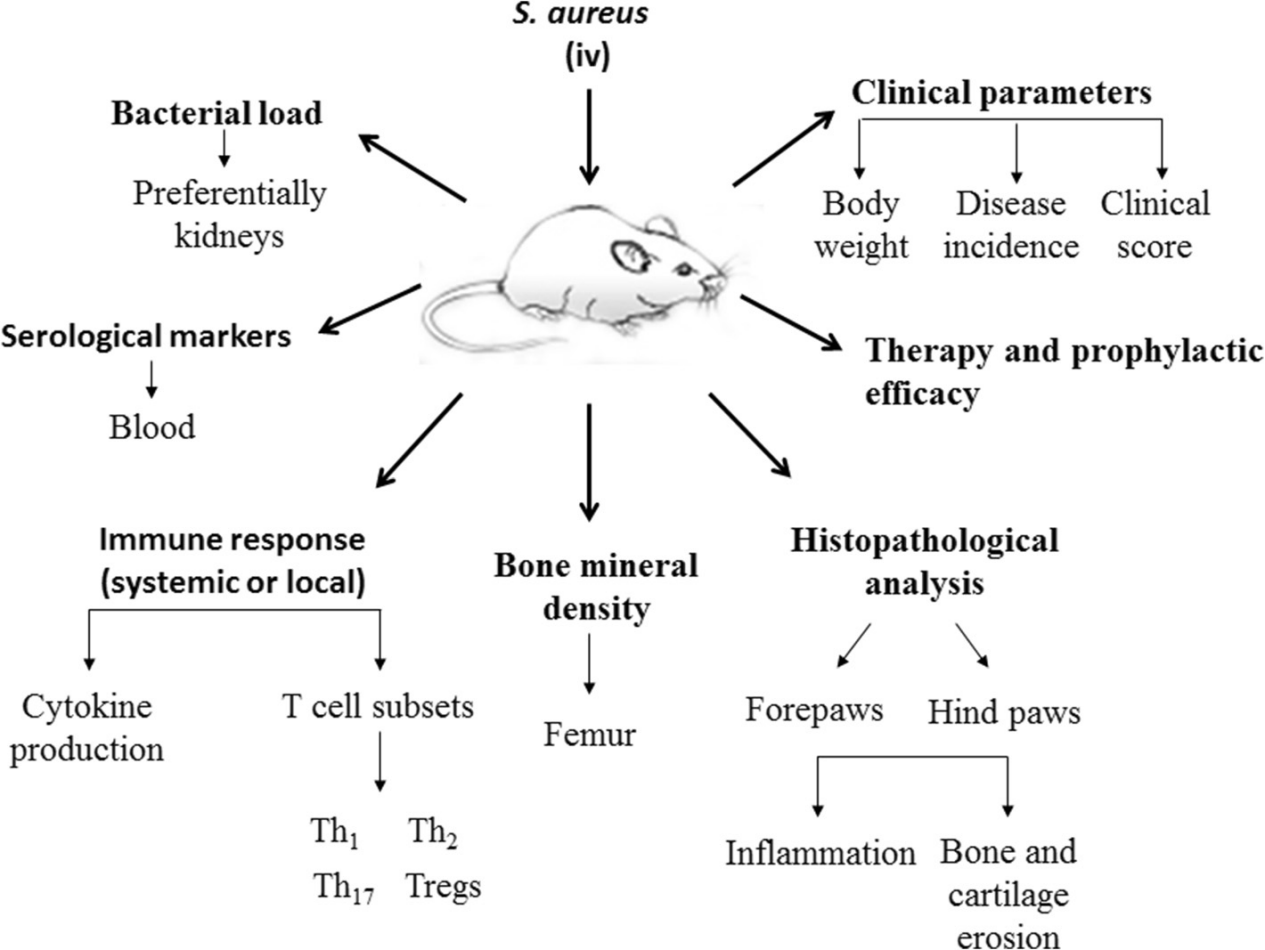
Schematic outline of the parameters that are most frequently analyzed in the murine SA experimental model.

There is a consensus that the best way to trigger SA is by the intravenous route. This procedure would better mimic the great majority of bacterial joint infections in humans that are believed to originate from the blood [11]. In our personal experience, the bacterial injection by the retro-orbital route is fast, highly effective and also generates a very homogeneous pathology. This model allows analysis of the disease development by daily joint inspection. Arthritis is defined by a visible joint erythema and/or swelling of at least one joint. To evaluate the intensity of arthritis, a clinical scoring (arthritic index) is carried out using a system where macroscopic inspection yields a score of 0–3 points for each limb (1 point = mild swelling and/or erythema; 2 points = moderate swelling and erythema; 3 points = marked swelling and erythema). The arthritic index is constructed by dividing the total score (number of arthritic limbs) by the number of animals used in each experimental group [40,41,52]. An illustrative micrograph of a clinical score 2 in C57BL/6 mice infected with an *S. aureus* strain is shown in Figure 1. This mouse model has been extensively used to assess bacterial virulence factors [17], host defense mechanisms [58], and the immunopathogenetic mechanisms that result in joint destruction [35]. The availability of this model is also revealing interesting alternatives in the fields of prophylactic and therapeutic procedures [60,61]. A schematic outline of the main parameters that have been analyzed in this model is presented in Figure 2. More detailed informations on the application of the murine model to investigate this pathology are available in the literature [35,58,62].

### Therapy

The speed and accuracy of SA therapy is critical to control disease aggravation. If SA is suspected, a blood sample and an aspiration of the joint should be performed before antibiotic administration. However, as soon as these procedures have been done and before the results are available, it is imperative to start treatment with broad-spectrum antibiotics [1,35]. A detailed description related to clinical management and treatment of human SA is outside of the scope of this review. However, highly informative data on this subject can be found in the literature [63-65]. There is also a consensus among the experts in the field that treatment should include the concomitant removal of any purulent material [66].

Even though it is well established that killing the bacteria is essential for controlling SA evolution, this treatment alone will not block the joint-destruction process. It has been described that after completing antimicrobial treatment, these patients recover around 46 to 50 percent of their original joint function [67]. This happens because much of the local destruction is generated by the immune response induced by the bacteria and their soluble products [35]. This realization prompted the association of antibiotics with substances able to counteract this exaggerated immune response. Experimental models have been extremely useful to test this concept. The severity of experimental SA was clearly down-regulated by the adjunctive association of corticosteroids with antibiotics [68,69]. The translation of this concept to treatment of human SA was confirmed [70]. This combined approach is clearly expanding in the treatment of SA and being thoroughly investigated with the help of the murine experimental model. Some of the most important findings in this field are summarized in Table 1.

**Table 1:** Experimental treatments in septic arthritis

**Treatment**	Control of arthritis development (*)	**Reference**
Inhibition of transcription factors NF-kB and mCoAP-1 alone or with antibiotics	No	[71]
Cloxacillin + phenyl-N-tert-butyl nitrone (antioxidant)	Yes	[72]
Cloxacillin + TNF inhibitor	Yes	[60]
Ampicillin + riboflavin (antioxidant)	Yes	[73]
Gentamicin + ascorbic acid	Yes	[74]
Estradiol	Yes	[75]
Azithromycin + riboflavin	Yes	[76]
Glutaminyl cyclase inhibitors	Yes	[77]

(*) Control of arthritis development was assessed by the following parameters: delayed disease development, less severe clinical manifestations or reduced swelling or synovitis.

## Conclusions

• Septic arthritis is a severe inflammatory disease of the joints triggered by an infectious agent.

• *Staphylococcus aureus* causes the most frequent and severe cases of septic arthritis.

• Joint destruction is determined by both bacterial and host factors.

• Mice experimentally infected with *S. aureus* strains are widely employed to study this disease.

Mice were manipulated in accordance with the ethical guidelines adopted by the Brazilian College of Animal Experimentation. All experimental protocols were approved by the local ethics committee for animal experimentation(CEEA), Medical School, Univ. Estadual Paulista (protocol number 291).

## Abbreviations

SA: Septic arthritis; SAg: Superantigen or superantigens; RA: Rheumatoid arthritis; ClfA: Clumping factors; FnBPs: Fibronectin binding proteins; PMNs: Polymorphonuclear granulocytes; TSST-1: Toxic shock syndrome toxin-1.

## Competing interests

The authors declare that there are no competing interests.

## Authors’ contributions

PMC and AS equally contributed to conceiving and writing this review. Both authors read and approved the final manuscript.
